# Notes on Obstetrics and Gynæcology

**Published:** 1876-08

**Authors:** E. N. Brush


ART. II.—*Notes on Obstetrics and Gynæcology.* By E. N. BRUSH, M. D.

I. Puerperal Eclampsia By SAMUEL C. BUSEY; M. D. (*Philadelphia Medical Times*, Aug. 5. 1876.

Dr. Busey, in this article, which is an abstract from his address as chairman of the Section on Obstetrics of the American Medical Association, refers to the pathology of the disease. He refers at the outset to the fact that primiparæ and plural pregnancies are more liable to convulsions than multiparæ. From this fact and from the fact that "depletion of the gravid womb is the most certain way of terminating the convulsive seizures," he thinks too much prominence has been given to the mechanical theory of causation—obstructive hyperæmia of the kidneys. While he acknowledges the force of the hypothesis he cannot accept its absolute verity. An apparent contradiction to the mechanical theory, lies in the fact that it is the gravid uterus and not every other abdominal tumor, which is associated with albuminuria. He thinks that pregnancy, not the period of utero-gestation, is the essential factor. The cause lying in the altered relation, not of the parts anatomically concerned, but of the functions of the animal ecomomy. He says:

"During pregnancy the mass of blood is augmented, its constituent fibrin is increased, the albumen is diminished, the number of red blood-corpuscles is reduced (most markedly so during the later months); its temperature is elevated, the proportion of solids lessens and the quantity of water increases with the progress of gestation, the normal relation which exists between the fibrin and water becomes disturbed, there is hypertropy of the left ventricle, the heart becomes stronger, arterial tension (especially in the primiparæ) is increased, and during labor the blood-pressure (Fritsch), both arterial and venous, rises, while a uterine contraction is present. Thus, conditions favoring fibrin-separation and congestions are present to a remarkable degree, and various viscera—brain, heart, lungs and kidneys—may be temporarily congested. There are also added and retained effete products, and consequent increased strain upon the kidneys. In the primiparæ the vascular apparatus (Harcourt Barnes*) is not adapted, and in many pregnant women the assimilation of nutriment is inadequate to the added physiological work, and the tension of the cerebral vessels, which increases with the process of gestation, attains its maximum (Madden†) during parturition, when convulsions most frequently occur. There is, in addition an increase of nerve force, irritation of the pneumogastric, and a nervous sensitiveness especially characteristic of pregnancy.

The evacuation of the gravid uterus is followed by an engorgement of the abdominal veins, which had been more or less ob-

*Amer. Jour. Obst., vol. viii, p. 719.
†Obst. Jour. Great Britain and Ireland, vol. ii, p. 239.

structed by the pressure of the enlarged organ. This abstraction of the blood from the thoracic organs and from the brain, harmless as it is in most cases of parturition, and salutary as it proves to be in a majority of cases of convulsions, may result in such a condition of cerebral anæmia, enfeebled and irregular cardiac action, and imperfect decarbonization of the blood, as to become, in conjunction with the deteriorated condition of the blood-mass, the immediate and exciting cause of post-partum convulsions.

The condition of the blood during pregnancy, which is aggravated by the loss of albumen, simulates anæmia, yet the condition of the system is that of physiological plethora, due to the increment of the blood-mass. During pregnancy and during labor the brain may contain a redundancy of this impoverished and deteriorated fluid, and yet be insufficiently nourished. The sudden engorgement of the abdominal veins after delivery, may withdraw from the cranical cavity the requisite amount of fluid. In both instances the brain is anæmic, in one case containing an excess, in the other a deficiency, of the altered and toxæmic blood."

It is these departures from the state of health, due to pregnancy; he thinks we must look for the predisposing and proximate causes of puerperal convulsions. He refers in conclusion, to some additional facts which have come to our knowledge. He says that the once accepted theory that convulsions were occasioned by a determination of blood to the head, gains some force from the points of resemblance pointed out by Prof. Walley, between the cerebral circulation in woman and the cow, to whom, parturient apoplexy and convulsions are mainly confined. These points of resemblance are in connection with the distribution of the internal cartoid artery, the formation of the basilar artery and the circle of Willis, which favor a larger and more direct supply of blood to the brain. If these researches should be correct, we have then, during pregnancy, a peculiar condition of the blood, increased arterial tension, augmented blood-pressure and an anatomical arrangement of the vascular supply, which favor intercranial congestion. To these he would add, toxæmia, from destruction of the red blood-corpuscles, and retained effete products from renal congestion; malnutrition, from loss and consumption of albumen and from an inadequate supply of nutriment; deficient consumption of oxygen, from diminution of muscular action.

The paper concludes with an account of the observations of Bourneville and others upon the elevation of temperature in puerperal eclampsia. Bourneville makes the following conclusions:

"1. During the eclampsic state the temperature is raised from the outset of the attack to its termination.

"2. In the intervals of the attacks the temperature remains elevated, and at the moment of the convulsions a slight accession takes place.

"3. If the eclampsic condition is about to terminate in death, the temperature continues to augment, and reaches a very elevated figure; if, on the contrary, the attacks diminish and the coma ceases in a definite manner, the temperature lowers progressively and returns to the normal standard."

After a brief consideration of the therapeutical indication of these facts, Dr. Busey concludes: "I will advance a step further, and submit the proposition that the various methods of preventive treatment owe their efficacy to their effects upon the blood mass and blood-vascular system. As a rule these are directed to the promotion of digestion, whereby the loss of albumen is replenished, and to the diminution of the hydræmia, by catharsis, diuresis, or diaphoresis."

II. A New Device for Securing Union in Staphyloraphy, Vesico-Vaginal Fistula, etc. By A. SCHEIBENZUBER, M. D. (*Ohio Medical and Surgical Journal*, Aug., 1876.)

The "Device" here described consists in passing the thread or wire through the edges of the fistula. allowing the edges of the holes to cicatrize and then pare the edges of the fistula and tighten the sutures. The object of allowing the needle holes to cicatrize is to prevent the thread tearing out when the sutures are drawn tight. As the inventor of this ingenious device does not report a case illustrating its use in vesico-vaginal fistula, we presume his knowledge of its merits in such cases is purely theoretical, practically he would find that the small needle holes, whose edges were cicatrized, would give him as much trouble as the original fistula, he would only be multiplying his difficulties, in most cases. In an operation for staphyloraphy, it might be of value, but we think that the section of the tendons of the levator and tensor palati muscles would obviate all difficulty in most cases from the thread tearing out.

III. Two cases of Pessary Impacted in the Vagina for more than a year. By GEO. BUCHANAN, M. A., M. D., (*Obstetrical Journal, Great Britain and Ireland*, July, 1876. Incarcerated Pessary. By DR LYMAN, in Proceedings of Boston Obstetrical Society. (*Boston Medical and Surgical Journal*, August 17, 1876.

The first case of Dr. Buchanan's was in a lady aged forty-eight. The pessary had been introduced nearly five years previously for prolapsed uterus. The patient was put under chloroform and after considerable effort the pessary was extracted. One of its limbs had ulcerated through the outer wall of the vagina, into the bladder, and was coated with a quarter of an inch of urinary deposit. Case second was a lady aged sixty-one, over a year previous to her being seen by Dr. Buchanan, a modification of Hodges' pessary had been introduced to support the uterus. The ring of the pessary was found to have ulcerated into the lateral vaginal wall to the depth of an inch, the ulcerated edges had fallen together and united, making it necessary to use the scalpel in removing the instrument. The reporter of these cases makes some very pertinent remarks upon not trusting too much to the "common sense" of the patient in cases like these, explicit directions should always be given to patients entrusted with the charge of any instrument or mechanical appliance. He advocates in prolapsus in women past the child-bearing period, the operation for occlusion of the vagina as preferable to the use of pessaries.

Dr. Lyman's case had worn the pessary for five years, its removal was attended with pain and some hemorrhage. The cervix uteri was deeply ulcerated and there was some leucorrhœa. The upper half of the pessary was deeply imbedded in the posterior cul-de-cal, and was roughened and corrugated. In his remarks upon the case Dr. Lyman very truly remarked that pessaries were often introduced without necessity. Other physicians present were of the same opinion, and remarked that they frequently did more mischief than could be counterbalanced by the good they were intended to accomplish.

